# The association of Conscientiousness and Neuroticism on BMI and health behaviours: exploring the impact of Healthy Neuroticism

**DOI:** 10.3389/fpsyg.2025.1634465

**Published:** 2025-10-01

**Authors:** Zandra Overgaard Pedersen, Kathrine Sørensen, Bettina Ewers, Jesper Dammeyer

**Affiliations:** ^1^Department of Diabetes Care, Copenhagen University Hospital - Steno Diabetes Center Copenhagen, Herlev, Denmark; ^2^Department of Psychology, University of Copenhagen, Copenhagen, Denmark; ^3^The National Research Centre for the Working Environment, Copenhagen, Denmark

**Keywords:** big five personality traits, neuroticism, conscientiousness, healthy neuroticism, BMI, physical activity, diet awareness

## Abstract

**Background:**

Existing research in personality traits provides evidence of associations between Conscientiousness, Neuroticism and Body Mass Index (BMI), with a growing interest in the concept of Healthy Neuroticism. However, the associations are not fully understood, and the mitigating role of health behaviours remains insufficiently investigated.

**Methods:**

This cross-sectional study investigated associations between Neuroticism, Conscientiousness, including high and low scores, Healthy and Unhealthy Neuroticism with BMI, and the mediating effects of physical activity and caring about having a healthy and nutritious diet, in a national sample of 21,619 adults. Personality traits were assessed using the Big Five Inventory-10, and health behaviours via self-reported physical activity and attitudes towards a healthy and nutritious diet. Statistical analyses were conducted using Hayes PROCESS macro for mediation with 5,000 bootstrap samples.

**Results:**

The results demonstrated that Neuroticism and Unhealthy Neuroticism were positively associated with BMI, whereas Conscientiousness and Healthy Neuroticism showed negative associations. Health behaviours both fully and partially mediated the relationship between personality traits and BMI.

**Conclusion:**

These findings provide evidence for Healthy and Unhealthy Neuroticism in relation to BMI and health behaviours. Furthermore, findings demonstrate associations between Neuroticism, Conscientiousness and BMI, with physical activity, and diet awareness acting as mediators. Stratified analysis suggests that the role of educational level in these relationships likely reflects underlying differences in health behaviours.

## 1 Introduction

It is well-established that health behaviours contribute to the risk of developing chronic diseases such as cancer, diabetes and cardiovascular diseases (Formica et al., 2020; American Diabetes Association Professional Practice Committee, 2021). This has led to a growing emphasis on individualised treatment to support the prevention of chronic diseases and clinical outcomes (Davies et al., 2022; Cefalu et al., 2024). However, providing support that aligns with individualised needs requires a comprehensive effort to identify which interventions that are relevant to each individual’s unique circumstances. Current approaches to health behaviour interventions are often population-based and tend to overlook individual differences, especially in terms of psychological factors, specific needs, and personal preferences. It has been highlighted that these challenges need to be addressed in order to cluster high-risk individuals into subgroups for improved risk stratification and prediction of risk factors, to improve healthcare strategies and to support clinicians in targeting and tailoring interventions to individual needs (Nolan et al., 2022). This would enable the healthcare system to more effectively address those with the greatest need and facilitate early improvements through tailored interventions for increasing physical activity, promoting healthier dietary habits, and supporting obesity management.

### 1.1 Neuroticism, Conscientiousness and body mass index

One of the great public health challenges in today’s society is the rising prevalence of overweight and sedentary behaviour. Overweight is a multifaceted condition that contributes to a wide array of chronic diseases, including cancer, diabetes and cardiovascular diseases (American Diabetes Association Professional Practice Committee, 2023; Bianchini et al., 2002). The causes of obesity are complex and multifaceted, with various mediating factors. Individual differences can explain some of the variations in overweight across individuals. In recent decades, research has established associations between the Big Five personality traits, health behaviours and clinical outcomes (Turiano et al., 2015; Sutin et al., 2011; Wimmelmann et al., 2018; Jokela et al., 2013; Lunn et al., 2014). Extraversion, Agreeableness, and Openness have been associated with health in previous research, but these associations tend to be weaker and less consistent (Shim et al., 2014; Sutin et al., 2011). Therefore, focuses this study on Neuroticism and Conscientiousness, as they show more consistent associations with health, likely due to their relevance for emotional reactivity and self-regulation (Sutin et al., 2011). Neuroticism reflects the individual’s emotional stability and resistance to pressures from the surrounding environment. High Neuroticism scores indicate stronger reactions to negative life events and stressors (Simonsen et al., 2017), whereas individuals with low Neuroticism scores are in general more emotionally stable and less reactive to stressful conditions (Dammeyer & Zettler, 2018; Pedersen et al., 2024). Conscientiousness represents the individual’s competence to be organised, goal-oriented, the degree of work ethic attitude and attention to details (Dammeyer & Zettler, 2018; Pedersen et al., 2024). Individuals with low Conscientiousness scores tend to be less focused on long-term goals, more impulsive, and may struggle with motivation and following through on commitment (Dammeyer & Zettler, 2018; Pedersen et al., 2024). A growing body of evidence suggests that Conscientiousness is positively associated with health behaviours and health-related outcomes, whereas Neuroticism is negatively associated (Atherton et al., 2014; Friedman et al., 2014; Bogg and Roberts, 2004; Weston et al., 2015). High Neuroticism has been associated with higher BMI, greater weight gain and increased weight fluctuations over time, whereas high Conscientiousness appears to have a protective effect against a high BMI (Sutin et al., 2011; Jokela et al., 2013; Sutin and Terracciano, 2016; Sutin and Terracciano, 2017; Sutin et al., 2015; Vainik et al., 2019). However, the underlying mechanisms remain insufficiently investigated. In particular, evidence on how health behaviours mediate these associations, and whether such pathways vary by socio-demographic factors such as educational level, remains limited (Turiano et al., 2018; Hampson et al., 2007). Even though BMI is widely used as a proxy for health, it has been noted to have limitations, particularly in distinguishing between fat mass, muscle mass, and metabolic health, and should therefore be interpreted with caution (Franco et al., 2025).

### 1.2 Physical activity and eating behaviours

Behavioural determinants, including physical activity, dietary habits and meal patterns have been found to explain approximately 50% of the association between Neuroticism, Conscientiousness and BMI (Sutin and Terracciano, 2016). This highlights the need for increased focus on behavioural determinants such as eating behaviour and physical activity to reduce the increasing prevalence of overweight. Studies investigating associations between physical activity and the Big Five personality traits have found that Neuroticism is negatively associated with physical activity (Tolea et al., 2012; Rhodes and Smith, 2006; Sutin et al., 2016; Wilson and Dishman, 2015), whereas individuals with high Conscientiousness tend to be more physically active and engage in less sedentary behaviour (Sutin et al., 2016). Research examining exercise intensity preferences indicates that individuals with high Neuroticism tend to prefer low-intensity exercise activities, whereas those with high Conscientiousness are more likely to prefer high-intensity exercise (Hagan and Hausenblas, 2005), which has several health-related benefits.

Research on the Big Five personality traits and eating behaviour shows that high Neuroticism and low Conscientiousness are associated with emotional eating (Elfhag and Morey, 2008), whereas low Neuroticism and high Conscientiousness are associated with restrained eating (Elfhag and Morey, 2008). Other research has demonstrated that Neuroticism is associated with a preference for salty foods, sweets, a lower intake of fruits and vegetables, higher consumption of sugar and saturated fats (Esposito et al., 2021), and a greater preference for convenience food (Mõttus et al., 2013). Individuals with low Neuroticism are in general better at resisting food cravings and binge eating (Sutin et al., 2011). Additionally, a preference for the Mediterranean diet and greater health awareness have been found to be positively associated with Conscientiousness (Mõttus et al., 2013). Overall, an individual’s personality trait appears to influence dietary choices and diet-related awareness.

### 1.3 Healthy and Unhealthy Neuroticism

The literature presents conflicting findings regarding the association between Neuroticism and health-related outcomes. While high Neuroticism is often associated with adverse health behaviours, some studies suggest it may also be associated with reduced mortality risk, and lower levels of inflammation markers, potentially mediated by increased physical activity (Weiss and Costa, 2005; Graham et al., 2018). These contrasting results of Neuroticism have led to the theory of Healthy Neuroticism (Friedman, 2000). Healthy Neuroticism is not defined consistently across studies, some researchers have investigated Healthy Neuroticism as the interaction of socioeconomics factors and sex on Neuroticism (Hagger-Johnson et al., 2012; Graham et al., 2020), whereas the majority of studies investigate the interaction of Neuroticism and Conscientiousness. Studies indicate that individuals with high Neuroticism and low Conscientiousness, are more likely to engage in undesirable health behaviours, such as smoking, exhibit higher levels of the inflammation marker interleukin 6 (IL-6), which may be driven by these undesirable behaviours, compared to individuals who score high on both Neuroticism and Conscientiousness (Healthy Neuroticism) (Weston and Jackson, 2014; Turiano et al., 2013). A series of three separate papers investigated the impact of Healthy Neuroticism on longevity, chronic diseases, and health behaviours (Graham et al., 2020; Turiano et al., 2020; Weston et al., 2020). One of the papers reported that high Conscientiousness may protect against the risk of smoking among individuals with high Neuroticism. It also found that individuals high in Neuroticism and low in Conscientiousness were more likely to be physically inactive (Graham et al., 2020). However, research on Healthy Neuroticism is still sparse, not least in the field of health behaviours, such as physical activity and dietary choices.

### 1.4 The current study

A better understanding of the complex relationship between Neuroticism, Conscientiousness, and health behaviours may be essential for effectively support individuals in improving BMI, physical activity, and dietary habits. While existing literature suggests that Neuroticism and Conscientiousness each play an important role in shaping health behaviours and weight-related outcomes such as BMI, less is known about their combined association with these factors. Additionally, personality traits scores commonly follow a normal distribution, individuals with scores that deviate by one or two standard deviations (SD) above or below the mean may differ in relation to health behaviours and BMI compared to those within the average range. Exploring how high and low personality trait scores are associated with health behaviours and BMI may offer valuable insights, as individuals with high or low scores could represent high-risk subgroups with both greater support needs and a higher potential for positive change in areas such as physical activity, dietary habits, and weight management, with a particular need for tailored support to enhance adherence within these areas. The aim of the present study is to examine whether Neuroticism, Conscientiousness, including high and low scores, as well as Healthy Neuroticism and Unhealthy Neuroticism, are associated with BMI. Secondly, the study explores whether and how these associations are mediated by health behaviours, specifically physical activity, and caring about having a healthy and nutritious diet.

## 2 Methods

### 2.1 Participants

This cross-sectional study utilises data from the SHILD survey (Survey of Health, Impairment and Living Conditions in Denmark), which investigates current living conditions in Denmark (Kjaer et al., 2019). The survey is conducted by The National Research and Analysis Centre for Welfare. We used data from the 2016 wave of the survey (Amilon et al., 2016). By use of the personal identification number, participants were invited from a randomly selected sample of citizens aged 18–64, drawn by Statistics Denmark. The survey was sent out to 38,000 potential participants (Amilon et al., 2016) of whom 22,771 responded, corresponding to a response rate of 59.9%. Informed consent was obtained from all participants. All procedures in this research were in accordance with the ethical standards of the institutional and national research committee and with the 1964 Helsinki declaration and its later amendments or comparable ethical standards. The present cross-sectional study was approved by the General Data Protection Regulation (p. 2024–15623).

### 2.2 Personality trait measurement and psychometric properties

#### 2.2.1 Neuroticism and Conscientiousness

Neuroticism and Conscientiousness were assessed using a Danish translation of the BFI-10 questionnaire (Dammeyer, 2020). The BFI-10 questionnaire includes two items for each trait, each rated on a five-point Likert scale ranging from “strongly disagree” (=1) to “strongly agree” (=5) (McCrae and Costa, 2008). For each trait, a sum score was calculated, resulting in a score ranging from 2 to 10. The BFI-10 is considered reliable and valid (Soto and John, 2009).

#### 2.2.2 High Neuroticism and High Conscientiousness

High Neuroticism scores and High Conscientiousness scores were defined as scores one SD or more above the mean for each trait within this sample. The average Neuroticism score was 6.1 with a SD of 1.5. Individuals with a Neuroticism score ≥8 was therefore categorised to the High Neuroticism group. Similarly, the average Conscientiousness score was 4.7 with a SD of 1.6. Therefore, individuals with a Conscientiousness score ≥6 were allocated to the High Conscientiousness group. Individuals with high scores were coded 2, and the rest were assigned as 1. Dichotomised variables were included to investigate the potential impact of high scores, as prior research indicates that high scores may be more strongly associated with health outcomes than average scores (Sutin et al., 2011).

#### 2.2.3 Healthy Neuroticism and Unhealthy Neuroticism

Healthy Neuroticism and Unhealthy Neuroticism were similarly defined based on the sample mean and one SD of the study sample as criteria. Healthy Neuroticism included individuals high in Neuroticism scores and high in Conscientiousness scores [Neuroticism scores ≥8 (mean 6.1 plus one SD of 1.5) and Conscientiousness scores ≥6 (mean 4.7 plus one SD of 1.6)]. Unhealthy Neuroticism consisted of individuals high in Neuroticism scores and low in Conscientiousness scores [Neuroticism score ≥ 8 (mean 6.1 plus one SD of 1.5) and Conscientiousness scores ≤3 (mean 4.7 minus one SD of 1.6)]. Individuals meeting these criteria were coded 2, and those who did not were coded 1.

### 2.3 Measurement of health behaviours and BMI

#### 2.3.1 BMI

Participants self-reported their height and body weight. BMI was calculated as body weight in kilogrammes divided by height in metres squared (kg/m^2^; WHO, 2010).

#### 2.3.2 Physical activity

The survey included a question regarding level of physical activity: *“How many days per week are you physically active for at least 30 minutes per day?”* The response scale was 1 to 7, equal to the number of days per week respondents were physically active for at least 30 minutes. No distinction was made between physical activity at work or in leisure time. No missing values were reported for physical activity. However, 15 responses contained values >7 days, which were defined as outliers and removed in the analyses where physical activity where the mediator.

#### 2.3.3 Healthy and nutritious diet

One survey question asked to: “*To what extent do you care about that your diet is healthy and nutritious?*.” The response categories were recoded as: 1: “To a high extent”; 2: “To some extent”; 3: “To a lesser extent”; 4: “Not at all.” “Refuse to answer” and “do not know” were categorised as missing data and were not included in the analysis. A total of 21,162 responses were included in the analyses with healthy and nutritious diet as a mediator, after excluding 472 responses due to missing values.

## 3 Analytical procedures

To examine the study’s aims, several statistical analyses were performed using SPSS (version 28.0.1.0) with significance level of *p* ≤ 0.05. No inclusion or exclusion criteria were applied, as participants were randomly selected from the Danish Civil Registration System.

### 3.1 Descriptives

Descriptive statistics, including means, standard deviations and frequencies were performed to understand and summarise group characteristics.

### 3.2 Mediation analyses

**Figure 1:**
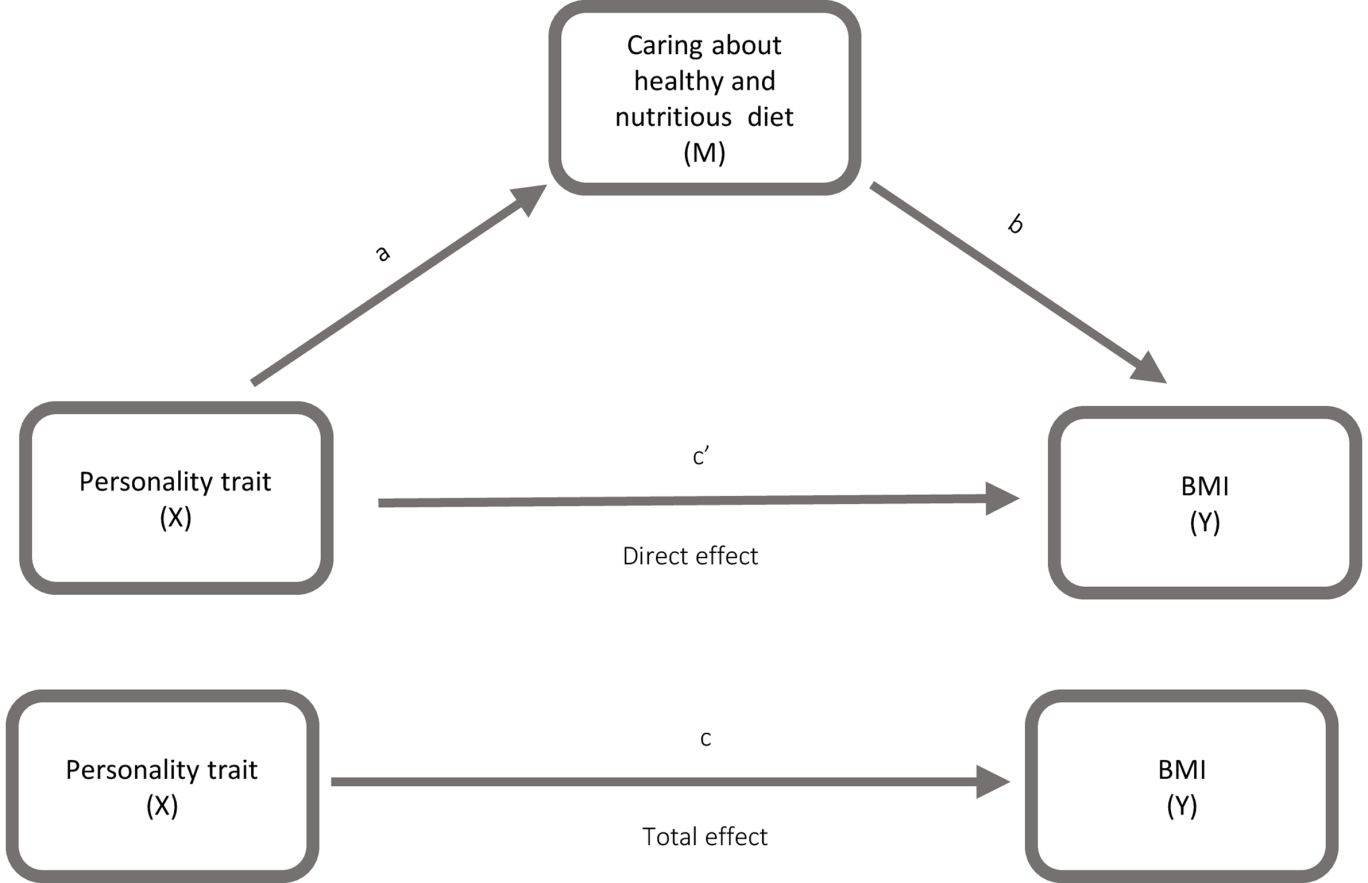
Model illustrating the mediating role of caring about a healthy and nutritious diet (M) in the association between personality traits (X) and body mass index (Y).

**Figure 2:**
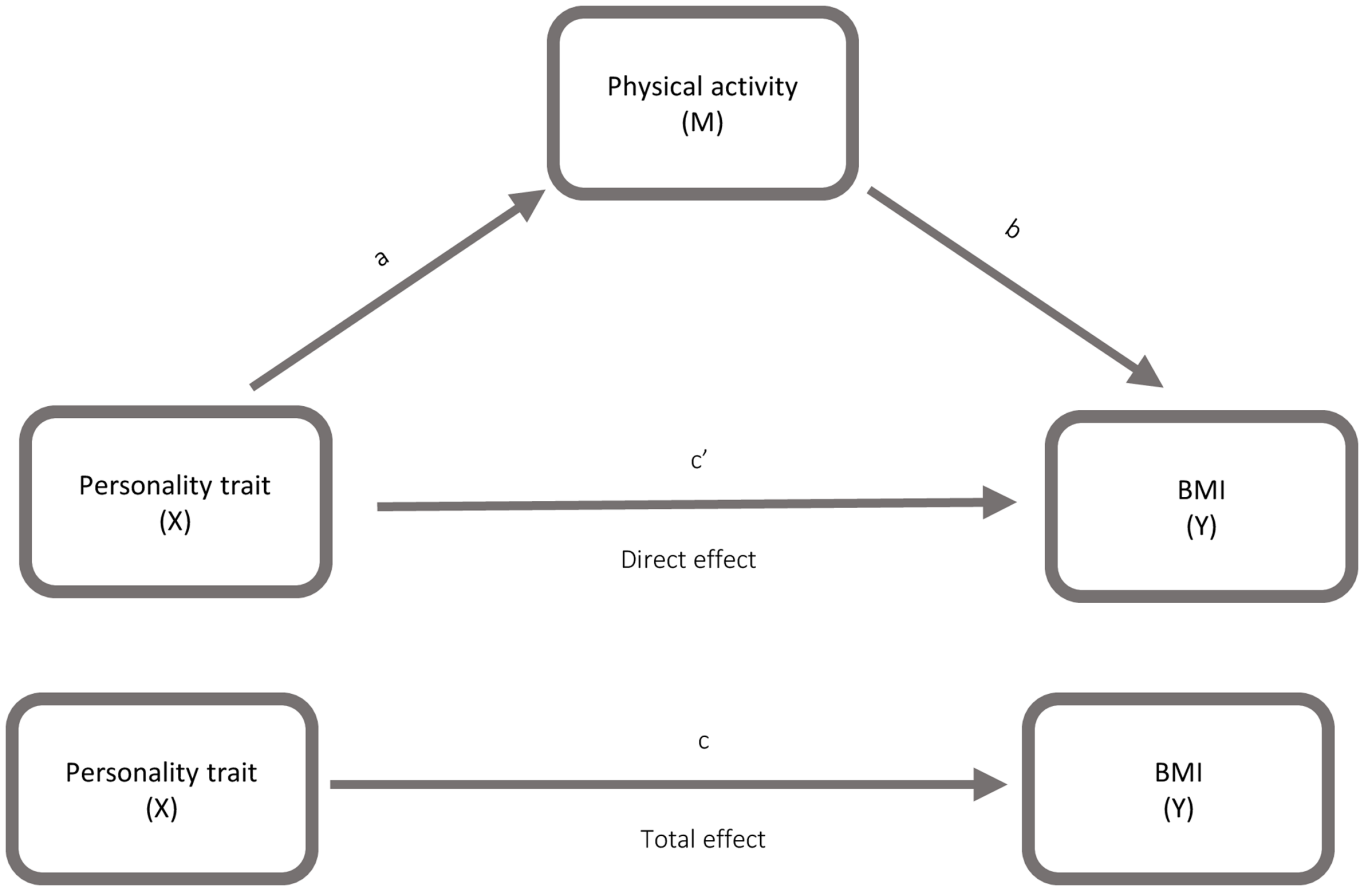
Model illustrating the mediating role of physical activity (M) in the association between personality traits (X) and body mass index (Y).

To investigate the association between personality traits and BMI, as well as the mediating role of physical activity and caring about having a healthy and nutritious diet, mediation analyses were conducted (See Figures 1, 2). Mediation analyses were conducted using Andrew F. Hayes PROCESS macro for SPSS. The models were run for each independent variable: Neuroticism, Conscientiousness, High Neuroticism, High Conscientiousness, Unhealthy Neuroticism and Healthy Neuroticism with BMI as the dependent variable. Mediators were (a) physical activity and (b) caring about having a healthy and nutritious diet. Only Neuroticism and Conscientiousness were entered into the statistical models. The remaining Big Five traits, Extraversion, Agreeableness and Openness, were excluded from the analyses. Model 4 of the PROCESS macro with 5.000 bootstrap samples, was applied for the mediation analysis. Data for Neuroticism, Conscientiousness, BMI, physical activity and caring about having a healthy and nutritious diet were analysed as continuous variables, whereas High Neuroticism, High Conscientiousness, Unhealthy Neuroticism and Healthy Neuroticism were analysed as dichotomous variables.

### 3.3 Stratification

Prior research has indicated that associations between personality traits and BMI differ between men and women in both strength and direction (Wimmelmann et al., 2018). Thus, stratified analyses by sex were conducted. The analyses were repeated for males and females separately. Stratified analyses were conducted for educational level, as education may moderate the strength or direction of the associations. Low educational level was defined as <3 years of higher education, and high educational level, defined as ≥3 years of higher education. Similar to the non-stratified analysis, mediation analyses were subsequently performed for each of the independent variables, using BMI as outcome variable and physical activity and if the participant cared about having a healthy and nutritious diet as mediators.

### 3.4 Assumption testing and outliers

To ensure that the assumptions for mediation analysis were met, data were initially examined for linearity, homoscedasticity and normality of residuals. To achieve normality in BMI, outliers were identified using the following criteria: values less than or equal to the 25th percentile (Q1) -1.5* Interquartile range (IQR) and values less than or equal to the 75th percentile (Q3) + 3 * IQR. The threshold of 3 times the IQR was chosen for the upper bound to retain BMI values up to 44.5, as using 1.5 would have excluded values above 36. Identified outlies were subsequently removed. A total of 1,137 values were identified as outliers based on BMI and removed from the dataset.

### 3.5 Power analysis

A *post hoc* power analysis using G*Power 3.1 (Faul et al., 2007) indicated that, with the given sample size (*N* = 21,619), the study was sufficiently powered (1–*β* > 0.99) to detect small effect sizes (f^2^ = 0.02) in the models, using a significance level of *α* = 0.05.

## 4 Results

### 4.1 Descriptives

Table 1 presents the participants characteristics including sex, age, BMI, educational level, and personality trait scores. The average age for the total sample was 43.4 years with a SD of 14.1. The average age was higher for both Unhealthy Neuroticism [mean age 45.2 years (SD 12.9)] and Healthy Neuroticism [mean age 48.5 years (SD 11.9)]. The total sample of 21,619 participants used in the mediation analysis of physical activity had an average BMI of 25.4 kg/m^2^ (SD 4.5), this where similar across all the subgroups based on personality traits, apart from Unhealthy Neuroticism where a slightly higher BMI of 26.4 kg/m^2^ was observed. Unhealthy Neuroticism presented with the lowest frequency of physical activity (mean 4.0 days, SD 2.1), whereas Healthy Neuroticism and Conscientiousness presented with the highest frequency of physical activity (mean 4.4 days, SD 2.1). Unhealthy Neuroticism presented with the highest mean of caring about having a healthy and nutritious diet (mean score 1.87, SD 0.7) and Healthy Neuroticism representing with the lowest mean of caring about having a healthy and nutritious diet (mean score 1.6, SD 0.6).

**Table 1:** Descriptive statistics.

Descriptives statistics					
	Total	High Neuroticism group	High Conscientiousness group	Unhealthy Neuroticism group*	Healthy Neuroticism group*
Total, (n)	21,619	3,152	6,872	478	1,372
Sex, M% /F%	46%/54%	39.1%/60.9%	38.8%/61.2%	44.4%/55.6%	33.7%/66.3%
Age, Years $\tilde{x}$ (SD)	43.4 (14.1)	46.8 (12.3)	46.0 (13.7)	45.2 (12.9)	48.5 (11.9)
BMI, kg/m^2^ $\tilde{x}$ (SD)	25.4 (4.5)	25.6 (4.4)	25.0 (4.2)	26.4 (4.9)	25.1 (4.1)
Physical activity $\tilde{x}$ (SD)	4.1 (2.1)	4.2 (2.1)	4.4 (2.1)	4.0 (2.1)	4.4 (2.1)
Caring about healthy and nutritious diet	1.8 (0.6)	1.74 (0.6)	1.75 (0.6)	1.87 (0.7)	1.6 (0.6)
Further educational level (%)					
No further education	44%	37%	42.1%	45.7%	35.8%
<3 y.	13.8%	15.1%	15.2%	11.7%	16.4%
3–4 y.	26.9%	30.8%	28.2%	26.8%	32.3%
≥5 y.	15.2%	16.4%	14.4%	15.5%	15.4%
Missing values	0.1%	0.2%	0.2%	0.2%	0.1%
Personality traits					
Neuroticism $\tilde{x}$ (SD)	6.1 (1.5)	8.5 (0.7)	6.3 (1.5)	8.5 (0.7)	8.6 (0.7)
Conscientiousness $\tilde{x}$ (SD)	4.7 (1.5)	5.1 (1.5)	6.4 (0.8)	2.7 (0.5)	6.5 (0.9)

M, Males; F, Females; *Healthy Neuroticism: individuals high in Neuroticism scores and high in Conscientiousness scores [Neuroticism scores ≥ 8 (mean 6.1 plus one SD of 1.5) and Conscientiousness scores ≥ 6 (mean 4.7 plus one SD of 1.6)]. Unhealthy Neuroticism: individuals high in Neuroticism scores and low in Conscientiousness scores [Neuroticism score ≥ 8 (mean 6.1 plus one SD of 1.5) and Conscientiousness scores ≤ 3 (mean 4.7 minus one SD of 1.6)].

### 4.2 Neuroticism and High Neuroticism

The total effect of Neuroticism on BMI, when Neuroticism was operationalised as a continuous variable was 0.11 (95% CI: 0.07; 0.15) in the main analysis (Table 2). When operationalised as High Neuroticism, we found the total effect for the subsample to be 0.27 (95% CI: 0.10; 0.44), (Table 2). The mediational effect of physical activity was very weak. For instance, the indirect effect of Neuroticism on BMI, through the mediator physical activity was −0.01 (95% CI: −0.01; −0.00), (Table 2). However, caring about having a healthy and nutritious diet appeared to mediate the association between Neuroticism and BMI, with an indirect effect of −0.05 (95% CI: −0.05; −0.04), as well as the association between High Neuroticism and BMI with an indirect effect of −0.11 (95% CI: −0.14; −0.08), (Table 3). Generally, the effects varied in the stratified analysis by sex and educational level, with the strata of males and low educational level showing stronger total effects of Neuroticism on BMI, and lower mediational effects of the mediators. Details can be found in Tables 2, 3.

**Table 2:** Hayes PROCESS mediations analysis: physical activity.

	Hayes mediation analysis (Bootstrap samples 5,000) of physical activity moderating the association between BMI and personality traits				
	Personality traits–physical activity	Physical activity–BMI	Personality traits–BMI direct effect	Personality traits–BMI; indirect effect	Personality traits–BMI; total effect
Coefficient and confidence intervals (CI 95%)					
All participants (*N* = 21,619)					
Neuroticism	0.03 (0.01; 0.05) *	−0.23 (−0.25; −0.20) *	0.12 (0.08; 0.16) *	−0.01 (−0.01; −0.00) *	0.11 (0.07; 0.15)*
Conscientiousness	0.08 (0.06; 0.10) *	−0.21 (−0.24; −0.19) *	−0.21 (−0.26; −0.18) *	−0.02 (−0.02; −0.01) *	−0.23 (−0.27; −0.19)*
High neuroticism	0.02 (−0.06; 0.09)	−0.22 (−0.25; −0.19) *	0.27 (0.11; 0.45) *	−0.00 (−0.02; 0.01)	0.27 (0.10; 0.44)*
High conscientiousness	0.34 (0.28; 0.40) *	−0.21 (−0.24; −0.19) *	−0.51 (−0.64; −0.38) *	−0.07 (−0.09; −0.06) *	−0.58 (−0.71; −0.45)*
Unhealthy neuroticism	−0.17 (−0.36; 0.02)	−0.22 (−0.25; −0.19) *	0.96 (0.55; 1.36) *	0.03 (−0.01; 0.08)	0.99 (0.59; 1.40)*
Healthy neuroticism	0.22 (0.11; 0.33) *	−0.22 (−0.25; −0.19) *	−0.20 (−0.45; 0.04)	−0.05 (−0.08; −0.02) *	−0.25 (−0.50; −0.01)**
Stratified on sex (Females; *n* = 11,666)					
Neuroticism	0.04 (0.01; 0.06) *	−0.30 (−0.34; −0.26) *	0.10 (0.04; 0.16) *	−0.01 (−0.02; −0.00) *	0.09 (0.03; 0.15) *
Conscientiousness	0.11 (0.09; 0.14) *	−0.29 (−0.33; −0.25) *	−0.15 (−0.22; −0.09) *	−0.03 (−0.04; −0.02) *	−0.18 (−0.24; −0.13) *
High neuroticism	0.03 (−0.07; 0.13)	−0.30 (−0.34; −0.25) *	0.17 (−0.07; 0.40)	−0.01 (−0.04; 0.02)	0.16 (−0.07; 0.39)
High conscientiousness	0.41 (0.33; 0.48) *	−0.29 (−0.33; −0.25) *	−0.31 (−0.48; −0.13) *	−0.11 (−0.15; −0.09) *	−0.42 (−0.60; −0.24) *
Unhealthy neuroticism	−0.18 (−0.42; 0.07)	−0.30 (−0.34; −0.26) *	0.78 (0.21; 1.36) *	0.05 (−0.02; 0.13)	0.83 (0.26; 1.41) *
Healthy neuroticism	0.21 (0.10; 0.34) *	−0.30 (−0.34; −0.25) *	−0.17 (−0.49; 0.15)	−0.06 (−0.10; −0.02) *	−0.23 (−0.55; 0.09)
Stratified on sex (Males; *n* = 9,953)					
Neuroticism	0.02 (−0.01; 0.04)	−0.15 (−0.19; −0.11) *	0.19 (0.13; 0.25) *	−0.00 (−0.01; −0.00) *	0.19 (0.13; 0.24) *
Conscientiousness	0.06 (0.03; 0.08) *	−0.14 (−0.18; −0.11) *	−0.21 (−0.27; −0.16) *	−0.01 (−0.01; −0.00) *	−0.22 (−0.28; −0.17) *
High neuroticism	0.01 (−0.12; 0.13)	−0.15 (−0.19; −0.11) *	0.65 (0.41; 0.90) *	−0.00 (−0.02; 0.02)	0.65 (0.40; 0.90) *
High conscientiousness	0.27 (0.17; 0.36) *	−0.14 (−0.18; −0.11) *	−0.51 (−0.69; −0.33) *	−0.04 (−0.06; −0.02) *	−0.55 (−0.73; −0.37) *
Unhealthy neuroticism	−0.16 (−0.45; 0.12)	−0.15 (−0.19; −0.11) *	1.21 (0.65; 1.77) *	0.02 (−0.02; 0.07)	1.23 (0.68; 1.79) *
Healthy neuroticism	0.27 (0.08; 0.47) *	−0.15 (−0.19; −0.11) *	0.13 (−0.25; 0.51)	−0.04 (−0.08; −0.01) *	0.09 (−0.30; 0.47)
Stratified high educational level (≥ 3 years of higher education; *n* = 9,052)					
Neuroticism	0.06 (0.03; 0.09) *	−0.26 (−0.30; −0.22) *	0.06 (−0.00; 0.12)	−0.02 (−0.03; −0.01) *	0.04 (−0.02; 0.11)
Conscientiousness	0.14 (0.11; 0.16) *	−0.24 (−0.28; −0.20) *	−0.24 (−0.30; −0.19) *	−0.03 (−0.04; −0.02) *	−0.27 (−0.33; −0.21) *
High neuroticism	0.13 (0.02; 0.24) *	−0.26 (−0.30; −0.21) *	0.08 (−0.15; 0.31)	−0.03 (−0.06; −0.01) *	0.05 (−0.18; 0.28)
High conscientiousness	0.44 (0.35; 0.53) *	−0.24 (−0.28; −0.20) *	−0.66 (−0.84; −0.48) *	−0.10 (−0.14; −0.08) *	−0.76 (−0.95; −0.58) *
Unhealthy neuroticism	−0.40 (−0.68; −0.12) *	−0.25 (−0.30; −0.21) *	1.05 (0.47; 1.62) *	0.10 (0.02; 0.18) *	1.15 (0.57; 1.72) *
Healthy neuroticism	0.40 (0.24; 0.56) *	−0.25 (−0.29; −0.21) *	−0.42 (−0.75; −0.10) *	−0.10 (−0.15; −0.06) *	−0.52 (−0.85; −0.19) *
Stratified low educational level (< 3 years of higher education; *n* = 12,437)					
Neuroticism	0.01 (−0.01; 0.04)	−0.21 (−0.25; −0.17) *	0.20 (0.14; 0.25) *	−0.00 (−0.01; 0.00)	0.20 (0.14; 0.25) *
Conscientiousness	0.05 (0.03; 0.07) *	−0.21 (−0.24; −0.17) *	−0.19 (−0.25; −0.14) *	−0.01 (−0.02; −0.01) *	−0.20 (−0.26; −0.15) *
High neuroticism	−0.07 (−0.18; 0.04)	−0.21 (−0.25; −0.17) *	0.51 (0.27; 0.76) *	0.02 (−0.01; 0.04)	0.53 (0.28; 0.77) *
High conscientiousness	0.27 (0.19; 0.35) *	−0.21 (−0.24; −0.17) *	−0.38 (−56; −0.20) *	−0.05 (−0.08; −0.04) *	−0.43 (−0.61; −0.26) *
Unhealthy neuroticism	−0.02 (−0.27; 0.23)	−0.21 (−0.25; −0.17) *	0.85 (0.29; 1.41) *	0.01 (−0.05; 0.06)	0.86 (0.29; 1.41) *
Healthy neuroticism	0.08 (−0.08; 0.24)	−0.21 (−0.25; −0.17) *	0.07 (−0.28; 0.43)	−0.02 (−0.05; 0.02)	0.05 (−0.30; 0.41)

*p-value <0.01, **p-value <0.05, coefficients are unstandardized.

**Table 3:** Hayes PROCESS mediations analysis: caring about having a healthy and nutritious diet.

	Hayes mediation analysis (Bootstrap samples 5,000) of caring about healthy and nutritious diet moderating the association between BMI and personality Traits				
	Personality trait–caring about healthy and nutritious diet	Caring about healthy and nutritious diet-BMI	Personality traits–BMI; direct effect	Personality traits–BMI; indirect effect	Personality traits–BMI; total effect
Coefficient and confidence intervals (CI 95%)					
All participants (*N* = 21,162)					
Neuroticism	−0.04 (−0.05; −0.04) *	1.1 (1.00; 1.19) *	0.16 (0.12; 0.20) *	−0.05 (−0.05; −0.04) *	0.11 (0.07; 0.15) *
Conscientiousness	−0.03 (−0.03; −0.02) *	1.03 (0.94; 1.13) *	−0.21 (−0.25; −0.17) *	−0.03 (−0.04; −0.02) *	−0.24 (−0.28; −0.20) *
High neuroticism	−0.10 (−0.12; −0.08) *	1.08 (0.10; 1.17) *	0.39 (0.22; 0.56) *	−0.11 (−0.14; −0.08) *	0.28 (0.11; 0.45) *
High conscientiousness	−0.11 (−0.13; −0.09) *	1.03 (0.94; 1.13) *	−0.47 (−0.60; −0.34) *	−0.11 (−0.14; −0.09) *	−0.58 (−0.71; −0.45) *
Unhealthy neuroticism	0.05 (−0.01; 0.11)	1.06 (0.97; 1.16) *	0.98 (0.57; 1.39) *	0.06 (−0.01; 0.12)	1.04 (0.63; 1.45) *
Healthy neuroticism	−0.17 (−0.21; −0.14) *	1.06 (0.97; 1.16) *	−0.08 (−0.32; 0.17)	−0.18 (−0.22; −0.15) *	−0.26 (−0.51; −0.02) **
Stratified on sex (Females; *n* = 11,561)					
Neuroticism	−0.04 (−0.05; −0.03) *	1.19 (1.05; 1.33) *	0.13 (0.08; 20) *	−0.04 (−0.06; −0.03) *	0.09 (0.03; 0.15) *
Conscientiousness	−0.02 (−0.03; −0.01) *	1.14 (1.00; 1.28) *	−0.17 (−0.22; −0.11) *	−0.02 (−0.03; −0.014) *	−0.19 (−0.25; −0.13) *
High neuroticism	−0.09 (−0.12; −0.06) *	1.17 (1.03; 1.31) *	0.26 (0.03; 0.05) *	−0.11 (−0.14; −0.07) *	0.15 (−0.08; 0.39)
High conscientiousness	−0.09 (−0.11; −0.07) *	1.14 (1.00; 1.29) *	−0.32 (−0.50; −0.14) *	−0.10 (−0.13; −0.07) *	−0.42 (−0.60; −0.24) *
Unhealthy neuroticism	0.06 (−0.01; 0.13)	1.16 (1.02; 1.30) *	0.76 (0.19; 1.33) *	0.07 (−0.02; 0.13)	0.83 (0.26; 1.41) *
Healthy neuroticism	−0.14 (−0.18; −0.10) *	1.16 (1.02; 1.30) *	−0.07 (−0.38; 0.25)	−0.16 (−0.22; −0.12) *	−0.23 (−0.55; 0.09)
Stratified on sex (Males; *n* = 9,601)					
Neuroticism	−0.03 (−0.04; −0.02) *	0.73 (0.61; 0.86) *	0.21 (0.16; 0.27) *	−0.02 (−0.03; −0.02) *	0.19 (0.13; 0.25) *
Conscientiousness	−0.02 (−0.03; −0.01) *	0.68 (0.55; 0.81) *	−0.21 (−0.26; −0.15) *	−0.01 (−0.02; −0.01) *	−0.22 (−0.27; −0.16) *
High neuroticism	−0.06 (−0.10; −0.02) *	0.71 (58; 0.84) *	0.73 (0.49; 98) *	−0.04 (−0.08; −0.02) *	0.69 (0.44; 0.94) *
High conscientiousness	−0.08 (−0.02; −0.05) *	0.68 (0.55; 0.81) *	−0.50 (−0.68; −0.32) *	−0.05 (−0.07; −0.03) *	−0.55 (−0.74; −0.37) *
Unhealthy neuroticism	0.06 (−0.03; 0.15)	0.69 (0.57; 0.82) *	1.33 (0.76; 1.89) *	0.04 (−0.03; 0.11)	1.37 (0.80; 1.95) *
Healthy neuroticism	−0.14 (−0.20; −0.08) *	0.70 (0.57; 0.81) *	0.17 (−0.22; 0.56)	−0.10 (−0.14; −0.05) *	0.07 (−0.31; 0.46)
Stratified high educational level (≥ 3 years of higher education; *n* = 8,988)					
Neuroticism	−0.03 (−0.04; −0.02) *	1.38 (1.24; 1.52) *	0.09 (0.03; 0.15) *	−0.05 (−0.06; −0.03) *	0.04 (−0.02; 0.11)
Conscientiousness	−0.04 (−0.05; −0.03) *	1.31 (1.17; 1.45) *	−0.23 (−0.28; −0.17) *	−0.05 (−0.07; −0.04) *	−0.28 (−0.34; −0.22) *
High neuroticism	−0.10 (−0.13; −0.07) *	1.37 (1.23; 1.51) *	0.20 (−0.03; 0.43)	−0.14 (−0.19; −0.09) *	0.06 (−0.17; 0.29)
High conscientiousness	−0.14 (−0.16; −0.11) *	1.32 (1.17; 1.46) *	−0.57 (−0.76; −0.40) *	−0.18 (−0.22; −0.14) *	−0.75 (−0.93; −0.57) *
Unhealthy neuroticism	0.11 (0.03; 0.20) *	1.36 (1.22; 1.50) *	1.01 (0.44; 1.58) *	0.15 (0.03; 0.28)	1.16 (0.58; 1.74) *
Healthy neuroticism	−0.17 (−0.22; −0.13) *	1.35 (1.21; 1.50) *	−0.28 (−0.60; 0.05)	−0.23 (−0.30; −0.17) *	−0.51 (−0.84; −0.18) *
Stratified low educational level (< 3 years of higher education; *n* = 12,051)					
Neuroticism	−0.03 (−0.04; −0.02) *	0.79 (0.66; 0.92) *	0.22 (0.17; 0.28) *	−0.02 (−0.03; −0.02) *	0.20 (0.14; 0.26) *
Conscientiousness	−0.02 (−0.03; −0.01) *	0.73 (0.06; 0.87) *	−0.19 (−0.25; −0.14) *	−0.01 (−0.02; −0.01) *	−0.20 (−0.26; −0.15) *
High neuroticism	−0.07 (−0.10; −0.03) *	0.77 (0.63; 0.90) *	0.60 (0.35; 0.84) *	−0.05 (−0.08; −0.02) *	0.54 (0.29; 0.79) *
High conscientiousness	−0.09 (−0.12; −0.07) *	0.74 (0.60; 0.87) *	−0.37 (−0.55; −0.20) *	−0.07 (−0.09; −0.05) *	−0.44 (−0.62; −0.26) *
Unhealthy neuroticism	0.02 (−0.06; 0.09)	0.75 (0.62; 89) *	0.90 (0.33; 1.47) *	0.01; (−0.05; 0.07)	0.91 (0.34; 1.49) *
Healthy neuroticism	−0.15 (−0.19; −0.10) *	0.76 (0.62; 0.89) *	0.15 (−0.21; 0.51)	−11 (−0.15; −0.07) *	0.04 (−0.32; 0.40)

*p-value <0.01, **p-value <0.05, coefficients are unstandardized.

### 4.3 Conscientiousness and High Conscientiousness

When Conscientiousness was operationalised as a continuous variable, the total effect on BMI in the main analysis was −0.23 (95% CI: −0.27; −0.19), (Table 2). When operationalised dichotomously as High Conscientiousness, the total effect was −0.58 (95% CI: −0.71; −0.45), (Table 2). The mediational effect of both mediators was overall weak for Conscientiousness. A statistically significant mediational effect was also observed for High Conscientiousness, with the highest indirect effect present in the strata’s based on high educational level, showing an indirect effect of −0.18 (95% CI: −0.22; −0.14) for caring about having a healthy and nutritious diet and −0.10 (95% CI: −0.14; −0.08) for physical activity. Overall, the total and indirect effects varied across the different strata, with higher educational level emerging as an important factor, showing stronger associations in both total and indirect effect. Overall, we found negative total effects across all analyses for both operationalisations and both mediational models.

### 4.4 Healthy and Unhealthy Neuroticism

Healthy Neuroticism and Unhealthy Neuroticism showed opposing total effects on BMI. Unhealthy Neuroticism was consistently positively associated with BMI across all analyses, with a total effect of Unhealthy Neuroticism on BMI of 0.99 (95% CI: 0.59; 1.40) in the main analysis (Table 2). No mediation effects were found in any analyses for Unhealthy Neuroticism, except for a partial complementary indirect effect of physical activity in the higher educational level stratum 0.10 (95% CI: 0.02; 0.18), (Table 2). The total effect of Healthy Neuroticism on BMI was statistically significant only in the main analysis −0.25 (95% CI: −0.50; −0.01), (Table 2) and within the strata of higher educational level across both mediation models. However, we did find mediational effects of both mediators for Healthy Neuroticism across the majority of analyses. Physical activity fully mediated the association between Healthy Neuroticism and BMI in the main analysis, with an indirect effect of −0.05 (95% CI: −0.08; −0.02). However, the strongest indirect effect of physical activity was observed in the strata of higher educational level −0.10 (95% CI: −0.15; −0.06), although this mediation was partial. Caring about having a healthy and nutritious diet appeared to mediate the association between Healthy Neuroticism and BMI, with an indirect effect of 0.18 (95%-CI: −0.22; −0.15) in the main analysis. The strongest effect was observed in the high educational strata −0.23 (95%-CI: −0.30; −0.17), where full mediation was evident in both analyses.

## 5 Discussion

The findings from this large sample demonstrate that Neuroticism and Conscientiousness are both significantly associated with BMI. In general, the associations between Neuroticism and BMI were stronger among men and individuals with lower educational levels. The total effect of Conscientiousness was consistently negatively associated with BMI, and this pattern remained when Conscientiousness was operationalised dichotomously as High Conscientiousness. Notably, the association appeared stronger within the higher educational level stratum. These associations were both fully or partially mediated by caring about having a healthy and nutritious diet and by being physical active. While the mediating effects were generally modest, they tended to be marginally more pronounced among individuals with higher educational levels.

Unhealthy Neuroticism was positively associated with BMI, with no evidence of mediation through physical activity or caring about having a healthy and nutritious diet, except for a partial complementary mediation by physical activity among individuals with higher educational levels. In contrast, Healthy Neuroticism was negatively associated with BMI. However, this was only observed in the main analysis and in the stratified analyses among participants with higher educational levels. These associations were partially mediated by physical activity and fully mediated by caring about having a healthy and nutritious diet, particularly in the higher education stratum.

### 5.1 Comparisons with previous studies

#### 5.1.1 Neuroticism and High Neuroticism

Aligning with previous research (Sutin et al., 2011) this study found a positive association between Neuroticism and BMI. However, in the analysis stratified by higher educational level, no associations were observed between neither Neuroticism or High Neuroticism and BMI. Previous studies have shown that educational level is associated with BMI (Halkjær et al., 2003) and that a higher educational level is associated with higher health literacy and maintaining a healthy lifestyle (Friis et al., 2016), which often expresses as a balanced BMI. Results from the present study indicate that educational level plays an important role in the relationship between Neuroticism and BMI.

The present study identified a modest positive association between Neuroticism and physical activity and found that physical activity marginally mediated the association between Neuroticism and BMI. This finding contrasts with a meta-analysis of 48,049 participants across 21 cohorts, which reported a small but consistent negative correlation between Neuroticism and physical activity (*r* = −0.11; range: −0.02 to −0.20), (Rhodes and Smith, 2006). The discrepancy raises the possibility of a Type I error in the current analysis, suggesting that the observed positive association may represent a false positive. Furthermore, this modest mediating effect indicates that physical activity alone does not fully account for the link between Neuroticism and BMI, highlighting the complexity of psychological and behavioural pathways influencing body weight. These results may inform tailored health interventions, suggesting that promoting physical activity in individuals with high Neuroticism might be beneficial, but insufficient on its own to impact BMI meaningfully.

Neuroticism and High Neuroticism were significantly negatively associated with caring about having a healthy and nutritious diet, indicating that these individuals reporting higher Neuroticism scores tend to place less importance to healthy eating. Previous literature has shown that higher levels of Neuroticism are associated with higher consumption of convenience foods and a lower awareness of healthy dietary practices (Mõttus et al., 2013; Mõttus et al., 2012). Although that associations between Neuroticism, food choices and attitudes towards a healthy diet are evident, an important mediating factor may be emotional eating. The emotional and behavioural patterns related to the characteristics of Neuroticism make it plausible to hypothesise that the positive association between Neuroticism and BMI, may be partly explained by a stress-related coping mechanism such as emotional overeating and preference for energy dense foods that provide temporary cessation and relief (Edwin Thanarajah et al., 2023). Future research could investigate specific behavioural patterns including the mediating role of emotional eating in the association between Neuroticism and BMI. This may help improve understanding of how individuals with high Neuroticism can be better supported.

#### 5.1.2 Conscientiousness and High Conscientiousness

In line with previous research (Jokela et al., 2013), Conscientiousness was negatively associated with BMI across all analyses, with the most dominant association observed in the High Conscientiousness subsample. Conscientiousness and High Conscientiousness were positively associated with physical activity across all analyses. Further, physical activity was found to mediate the association between Conscientiousness and BMI. Previous studies have suggested a reciprocal relationship between Conscientiousness and physical activity, where higher Conscientiousness predicts increased physical activity, and that physical activity, in turn, is associated with increases in Conscientiousness among women, indicating these factors mutually affect each other (Allen et al., 2017). Since our study is cross-sectional, we are not able to account for such potential reciprocal relationships.

Other studies have shown that men with high Conscientiousness consume less fast food and drink less alcohol (Wimmelmann et al., 2018). Additionally, higher Conscientiousness has been associated with a lower intake of carbohydrate-based food and meat, simultaneously with a higher intake of fish and plant-based food (Pfeiler and Egloff, 2020). These findings suggest that higher Conscientiousness may influence dietary choices in a healthier and more nutritionally advantageous direction. However, results from this study indicated a negative association between Conscientiousness and caring about having a healthy and nutritious diet. This apparent discrepancy may be explained by the fact that participants in our present study were asked about their general attitudes towards diet rather than their specific food intake.

#### 5.1.3 Healthy Neuroticism and Unhealthy Neuroticism

Mixed results have been reported in previous research for Healthy Neuroticism, with some studies finding significant associations (Graham et al., 2020; Weston and Jackson, 2014; Roberts et al., 2009), while others found no evidence, suggesting that Conscientiousness has no mitigating effect on Neuroticism (Turiano et al., 2020; Weston et al., 2019). These conflicting results may reflect, among the few existing studies, that some studies comprise of small sample sizes, divergent definitions of Healthy Neuroticism, and different analytical approaches across studies. In our study, the findings for Healthy Neuroticism and Unhealthy Neuroticism were clear. Findings for Healthy Neuroticism demonstrated that Conscientiousness mitigated the relationship between Neuroticism, physical activity, and BMI, especially among individuals with higher educational levels, while Unhealthy Neuroticism demonstrated the strongest association with BMI. Specifically, a positive association between Healthy Neuroticism and physical activity could be detected, but this association was insignificant in the stratified analysis for low educational level, whereas a positive association between Unhealthy Neuroticism and physical activity could only be detected in the stratified analysis for higher educational levels. Indicating that educational level, or factors associated with educational level, might be an essential factor in the associations between Healthy Neuroticism, Unhealthy Neuroticism, BMI and health behaviours. Therefore, future interventions might benefit from being tailored to account for educational background and related psychosocial resources, for example health literacy, as it seems to play a pivotal role (Friis et al., 2016). Further investigation in the construct Healthy and Unhealthy Neuroticism should be carried out to better understand different cut-off scores of Neuroticism and interactions with Conscientiousness scores.

### 5.2 Methodological considerations

The study has several notable strengths. First, it is based on a large sample, randomly selected by Statistics Denmark using national personal identification numbers. Furthermore, we examined the potential mediating roles of sex and educational level, allowing for a more nuanced understanding of how these factors may influence the associations between personality traits and BMI. However, several limitations apply for this study. First, due to the cross-sectional observational study design, causal relationships cannot be established. Second, the dietary question used was broad and nonspecific, failing to capture information about actual dietary intake. Third, the study relied on self-reported data, which poses the risk of social desirability bias, and other reporting inaccuracies, such as underreporting of body weight used to calculate BMI, and overreporting of health behaviours. Fourth, unmeasured confounding variables may explain part of the observed associations between personality traits and the outcome variables. Fifth, there is a potential for healthy volunteer bias, as individuals with more socioeconomically advantageous backgrounds are generally more likely to participate in survey-based studies. Sixth, this study focused solely on Neuroticism and Conscientiousness. While these traits show the most consistent associations with health outcomes, excluding the remaining Big Five traits may have resulted in unaccounted variance. This limits the ability to account for shared variance between the traits and may result in certain behaviours being misattributed to Neuroticism or Conscientiousness. Including all five traits in future analyses would allow for a nuanced attribution of effects. Seventh, the study used BMI as a proxy for metabolic health. Additional health indicators could have provided a more nuanced understanding of relevant associations. Eight, the generalisability of the findings is limited to a Danish context. Prior research suggests that sociocultural environments can moderate the relationship between Neuroticism and BMI, for example exposure to sociocultural environment in the United States has been shown to influence this association (Sutin et al., 2015). Indicating that the infrastructure of the society plays an imperative role and may have a shaping impact of dietary choices and physical activity habits, which limits our study’s findings to only reflect associations among the Danish population and the sociocultural norms related to the Danish society. Additionally, the associations found in this study may reflect the specific conditions of the time period in which data was collected, as national health strategies could have influenced the population. The SHILD survey was conducted in 2016, 4 years after The Danish Ministry of Health and Prevention introduced the “prevention packages” in 2012 (SST, 2013). This national initiative may have influenced population-level health behaviours and thereby introduced random variation or served as an unobserved confounding factor in the analyses.

### 5.3 Implications

A recent paper within the field of precision health emphasises that healthcare solutions should be tailored to the needs of diverse populations, addressing specific challenges and requirements these individuals face (Cefalu et al., 2024). Achieving this goal requires a multifaceted approach that addresses individual differences and identify those who do not benefit from the traditional healthcare strategies, particularly those who require the most attention and support. Graham et al. (2018) and Sutin et al. (2016) has earlier noted that for understanding the complexity of health related outcomes and associations with personality traits, individuals of significant interest are those who deviate markedly from the mean score (Sutin et al., 2016; Graham et al., 2018), which also might be individuals of special interest for the healthcare system. Understanding the emotional and behavioural response patterns of individuals high in Neuroticism is central to providing more effective and tailored support. Research has shown that individuals high in Neuroticism are more likely to receive healthcare advice from others, but have a counterproductive emotional and behavioural response to these advices (Tucker et al., 2006). Additionally, it has been suggested that emotional regulation difficulties is an factor in the relationship between Neuroticism and unhealthy behaviours, and that these should be addressed in interventional programmes (Singh, 2022). Such individuals may not benefit from the traditional health consultations provided by the health care system. If health interventions are tailored to the challenges that individuals high in Neuroticism meets, it may enhance adherence and facilitate optimal conditions for achieving health-related goals. For these individuals emotional and stress-related eating appears to be a significant barrier, suggesting that tailored interventions should incorporate components for emotional coping strategies and self-regulation. A higher intake of convenience foods has also been observed for individuals high in Neuroticism, underscoring the need for structured meal planning and education on simple, healthy meals. In contrast, individuals high in Conscientiousness are often more likely to adhere to health-promoting behaviours due to their long-term goal orientation. Effective support strategies for this group may involve reinforcing the long-term health benefits of sustained health behaviours and connections between daily actions and measurable health outcomes. In the current study, men with high Neuroticism, especially those with lower educational levels, were less physically active. Previous literature suggests, that men need different interventions and a different delivery of information than women for motivating them to sign up and effectively take part in the health programmes (White et al., 2011). Taken together, these findings support the importance of developing tailored health strategies for different subgroups, based on personality traits, educational level, and sex. Such strategies should acknowledge the varying health challenges, motivations, and behavioural patterns present across individuals to improve intervention reach, engagement, and effectiveness.

### 5.4 The role of personality traits in behaviour change

Emerging evidence in behavioural research highlights the potential of personality traits to enhance behaviour change by refining health interventions to the specific characteristics of the target population, which may improve behavioural changes, adherence, and improve the use of healthcare resources (Chapman et al., 2014). By aligning interventions with individuals’ specific needs, motivations, and self-regulatory tendencies, all factors shaped by personality traits, may enhance both engagement and effectiveness, as individuals are more likely to perceive the intervention as relevant and meaningful, thereby increasing the likelihood of behavioural change (Chapman et al., 2014). A recent study found that Conscientiousness and Healthy Neuroticism were associated with greater increases in daily steps during a five-week physical activity intervention period, when compared with the remaining Big Five traits (Stieger et al., 2020). These findings indicate that the efficacy of the intervention modality may depend on personality traits and trait specific preferences, which can be taken into account, in designing interventions for different sub-populations.

## 6 Conclusion

The results of this study provide evidence for the significance of Healthy Neuroticism and Unhealthy Neuroticism, suggesting that there is an ameliorated effect of Conscientiousness on Neuroticism in relation to BMI and health behaviours. Furthermore, findings provided evidence that Neuroticism and Conscientiousness are associated with BMI, and that these associations are mediated by physical activity and caring about having a healthy and nutritious diet. Stratified analyses showed that educational level has an impact on the associations between Neuroticism, Conscientiousness and BMI, when health behaviours are considered as mediators.

## Data Availability

The data supporting the conclusions of this article are not publicly available due to data sharing restrictions, but further information can be obtained from the corresponding author upon reasonable request.
